# Pre-dialysis acute care hospitalizations and clinical outcomes in dialysis patients

**DOI:** 10.1371/journal.pone.0209578

**Published:** 2019-01-16

**Authors:** Silvi Shah, Karthikeyan Meganathan, Annette L. Christianson, Anthony C. Leonard, Charuhas V. Thakar

**Affiliations:** 1 Division of Nephrology, Kidney CARE Program, University of Cincinnati, Cincinnati, Ohio, United States of America; 2 Department of Biomedical Informatics, University of Cincinnati, Cincinnati, Ohio, United States of America; 3 Department of Family and Community Medicine, University of Cincinnati, Cincinnati, Ohio, United States of America; 4 Division of Nephrology, VA Medical Center, Cincinnati, Ohio, United States of America; Kaohsiung Medical University Hospital, TAIWAN

## Abstract

**Background:**

Patients with chronic kidney disease (CKD), a precursor of end stage renal disease (ESRD), face an increasing burden of hospitalizations. Although mortality on dialysis is highest during the first year, the impact of pre-dialysis acute hospitalizations on clinical outcomes in dialysis patients remains unknown.

**Methods:**

We evaluated 170,897 adult patients who initiated dialysis between 1/1/2010 and 12/31/2014 with linked Medicare claims from the United States Renal Data System. Using logistic regression models, we examined the association of 2-year pre-dialysis hospitalization on the primary outcome of 1-year all-cause mortality. Secondary outcomes included 90-day mortality, type of initial dialysis modality and type of vascular access at hemodialysis initiation.

**Results:**

Mean age was 72.7 ± 11.0 years. In the study sample, 76.0% of patients had at least one pre-dialysis hospitalization. Compared to patients with no pre-dialysis hospitalization, the adjusted 1-year mortality was higher with pre-dialysis cardiovascular related hospitalization (odds ratio [OR], 1.63; 95% confidence interval [CI], 1.57–1.68), infection related hospitalization (OR, 1.51; CI, 1.45–1.57), both cardiovascular and infection hospitalization (OR, 1.91; CI, 1.83–1.99), and neither-cardiovascular nor-infection hospitalization (OR, 1.23; CI, 1.19–1.27). Additionally, the adjusted odds of hemodialysis vs. peritoneal dialysis as the initial dialysis modality were higher, whereas adjusted odds to initiate hemodialysis with an arteriovenous access vs. central venous catheter were lower in patients with any type of hospitalization.

**Conclusion:**

Pre-dialysis hospitalization is an independent predictor of 1-year mortality in dialysis patients. Reducing the risk of pre-dialysis hospitalization may provide opportunities to improve quality of care in ESRD.

## Introduction

Approximately 30 million adults in the United States have chronic kidney disease (CKD). CKD, which can be a precursor to end stage renal disease (ESRD), is associated with high burdens of mortality and cost of care [1–4]. Additionally, patients with ESRD are at a high risk of morbidity, and experience an annual mortality of 15–20% [4, 5]. Over recent years, the Center for Medicare and Medicaid Services (CMS) has reformed the payment system for ESRD programs with a focus on performance. Annual mortality rate, derived from patient characteristics at the time of dialysis initiation, is a common metric used to compare quality of care and determine reimbursements across dialysis facilities [6].

One of the major factors affecting morbidity and mortality after dialysis initiation is acute hospitalization. Several studies have examined the trends and causes of hospitalizations in ESRD patients receiving dialysis [7–9]. It is recognized that CKD is also associated with an increased risk of hospitalizations; and the risk of hospitalizations increases with worsening stages of CKD [10, 11]. It is both plausible and intuitive that patient factors and process of care factors during the pre-ESRD period will continue to influence outcomes after the initiation of dialysis. To that effect, studies have shown that patients receiving pre-ESRD nephrology care are more likely to choose home-based dialysis modalities, have arteriovenous (AV) access for hemodialysis, and experience improved survival [12–14]. On the other hand, despite the high risk of hospitalization in CKD patients, no studies have examined the effect of pre-ESRD acute care hospitalizations on dialysis outcomes. Hence, evaluating the impact of pre-ESRD hospitalizations on post-dialysis care could fill critical knowledge gaps by providing prognostic information at the time of dialysis initiation, and may create new opportunities to improve clinical care in this specific patient population.

We hypothesized that the types or frequencies of hospitalizations occurring prior to ESRD would influence dialysis outcomes. We tested this hypothesis by leveraging the unique strengths of the United States Renal Data Systems (USRDS), which is the largest comprehensive registry of ESRD patients, and linked Medicare claims information for two complete years prior to incident ESRD. We examined the primary outcome of all-cause 1-year mortality; and secondary outcomes of mortality at 90-days, choice of initial dialysis modality, and type of vascular access in hemodialysis subjects.

## Methods

### Designs, setting and participants

**Fig 1:**
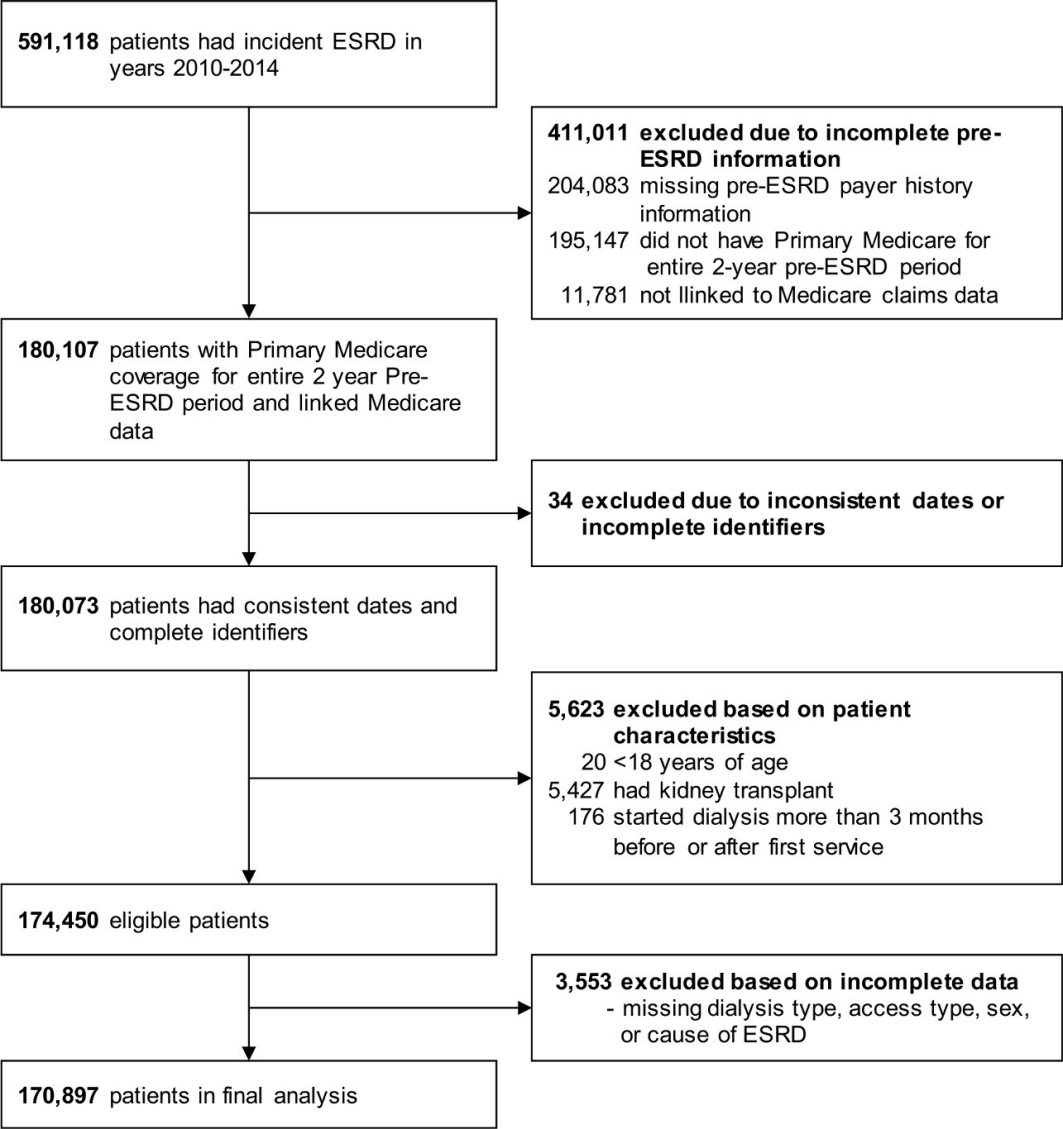
Flow chart describing the derivation of the study cohort.

We identified all patients with ESRD who initiated dialysis between January 1, 2010 and December 31, 2014 from the “back-casted” database USRDS, with Medicare Part A and Part B or Medicare Primary Other as the primary payer for the entire 2 years prior to dialysis initiation and linked Medicare claims data (n = 180,807), and followed them until December 31, 2015. We excluded patients younger than 18 years old, those with history of kidney transplant, those with inconsistent dates/identifiers, those that had started dialysis more than 3 months after or before date of first ESRD service due to discrepant or missing date of first dialysis treatment (n = 5,657), and those with missing information on type of dialysis, dialysis access, gender or cause of ESRD (n = 3,553). This resulted in 170,897 patients available for analysis in our final study sample. **Fig 1** shows the derivation of study cohort. The study was reviewed and deemed exempt by the University of Cincinnati institutional review board.

### Definitions of predictor, outcomes and covariates

The major predictor of interest for all outcomes was the presence or absence of a pre-dialysis acute care hospitalization. We searched the primary Medicare inpatient claims for all study participants during the complete two years preceding ESRD using International Classification of Disease 9^th^ revision Clinical Modification *(*ICD-9-CM) codes for discharge diagnoses of hospitalization for an infection or cardiovascular event by using a previously validated method **(S1 Table)** [15]. Our study design intended to capture pre-ESRD hospitalizations, and hence we counted and considered those hospitalizations that concluded pre-incident ESRD. Therefore, we did not count the index hospitalization, which may have occurred at the time of dialysis initiation. Major predictor of hospitalization status was evaluated in two ways. We evaluated five groups of patients by primary diagnosis of pre-dialysis hospitalizations: (1) cardiovascular hospitalizations, (2) infection hospitalizations, (3) both cardiovascular and infection hospitalizations, (4) neither cardiovascular nor infection hospitalizations, and (5) no hospitalization. We further evaluated five groups of patients by frequency of pre-dialysis hospitalizations: 0, 1, 2, 3, and ≥4. The primary outcome was 1-year all-cause mortality. Secondary outcomes were 90-day mortality, type of dialysis modality (hemodialysis vs. peritoneal dialysis [PD]) and type of vascular access during hemodialysis initiation (AV access vs. central venous catheter [CVC]). Additionally, we determined whether history of pre-dialysis hospitalization was associated with in-hospital dialysis initiation. In-hospital dialysis initiation was determined by comparing the date of first dialysis initiation against pre-ESRD and ESRD hospitalization dates. Patients were followed until the primary end point or end of the study follow-up period, whichever occurred earlier. “Medical Evidence Form”, CMS-2728 form was used to ascertain information on demographics, chronic comorbid conditions (congestive heart failure, atherosclerotic heart disease which included other cardiac disease, hypertension, diabetes mellitus, cancer, peripheral vascular disease, transient ischemic attack/ cerebrovascular accident, chronic obstructive pulmonary disease and amputation), laboratory data (serum albumin and serum hemoglobin), nursing home history, cause of ESRD, provision of transplant information, history of smoking, alcohol use, drug use, employment history, primary dialysis setting, functional status, and pre-dialysis nephrology care. We defined poor functional status was defined by any of three co-morbid conditions specified in form CMS-2728 –inability to ambulate, inability to transfer, or need of assistance with daily activities [16]. The primary cause of death was obtained from form CMS-2746 and divided into the following: cardiovascular disease, infections, malignancy, withdrawal of dialysis and others/unknown [5].

### Statistical analyses

Distributions of categorical variables are expressed as frequencies or percentages, and continuous variables are described using mean and standard deviation (SD). Bivariate comparisons of subjects who did or did not have a pre-dialysis acute hospitalization were made using t tests, chi-squared tests, and Fisher exact tests for continuous and categorical variables as appropriate. Statistical significance was set at a two-tailed p-value of 0.05, unadjusted for multiple tests. We conducted multivariable regression analyses to examine the effects of pre-dialysis hospitalizations and their causes and frequencies vs. no acute hospitalization, on 1-year mortality, and secondary outcomes. In addition, the primary outcome of 1-year mortality was analyzed using a multivariable Cox proportional analysis censored for 1-year of follow up, loss to follow-up and recovery of renal function. Other covariates included in the regression model specification process were demographic variables, comorbidities, laboratory data, nursing home history, ESRD cause and access to transplant information. Multivariable models were constructed by applying backwards elimination to a set of candidate predictors until all predictors remaining had *P* values less than 0.05, adjusted for other predictors left in the model. The risk estimates were expressed as odds ratios (ORs), hazard ratios (HRs) and their 95% confidence intervals (95% CIs). Kaplan-Meier methods were used to construct unadjusted survival curves, and the survival rates of different groups were compared using a log-rank test. Data were analyzed using SAS (SAS Institute, Cary, NC).

## Results

The sample was 54.4% male and 28.1% octogenarian, with a mean age of 72.7 years (SD, 11.0). The frequencies of hypertension, congestive heart failure, or diabetes mellitus among the patient population were 87.3%, 41.9%, and 56.8%, respectively. Of the study cohort, 76.0% had at least one pre-dialysis hospitalization. Among the causes of pre-dialysis hospitalization, 28.4% of patients experienced cardiovascular hospitalization, 12.3% had infection hospitalization, 11.3% had both cardiovascular and infection hospitalization and 24.5% had neither cardiovascular nor infection hospitalization. With regards to frequency of pre-dialysis hospitalization, 24.1% experienced 1 hospitalization, 17.2% had 2 hospitalizations, 11.5% had 3 hospitalizations, and 23.7% had ≥ 4 hospitalizations. For those with history of hospitalization during 2-year pre-ESRD period, mean length of stay in the hospital during all hospitalizations was 22.7 days (SD, 25.7). Hemodialysis was the initial dialysis modality in 94.3% of patients; its frequency was 91.2% in patients who did not have a pre-dialysis hospitalization, compared with 95.3% in those who did have a pre-dialysis hospitalization (*P* < 0.001). AV access was the vascular access used for initiation of hemodialysis in 20.0% of patients. Patients with a pre-dialysis hospitalization had a lower frequency of AV access use for dialysis initiation than without pre-dialysis hospitalization (17.0% vs. 28.0%, p<0.001). Rates of no pre-dialysis nephrology care were higher in the group with hospitalization as compared to patients without hospitalization (24.9% vs. 21.6%, p<0.001). More patients with pre-dialysis hospitalizations were females and had CHF as comorbidity. Patients with a pre-dialysis hospitalization had higher rate of low albumin < 3.5 (49.6% vs. 41.7%, p<0.001) and higher rates of low hemoglobin < 11 (72.2% vs. 68.7%, p< 0.001). **Table 1** shows comparison of patient characteristics across those who had pre-dialysis hospitalization vs. those who did not.

**Table 1:** Baseline characteristics of the study cohort stratified by presence and absence of pre-dialysis hospitalization.

Variable	All	No Pre-dialysis Hospitalization	Any Pre-dialysis Hospitalization	P Value
Number of Subjects, n (%)	170,897	40,214 (24.0)	130,683 (76.0)	
Age (year)	72.7 (11.0)	73.3 (10.4)	72.5 (11.1)	<0.001
Age category, %				<0.001
18–29	0.2	0.1	0.2	
30–39	1	0.7	1	
40–49	2.8	2.3	2.9	
50–59	7.6	6.5	8	
60–69	21.2	20.9	21.2	
70–79	39.2	41	38.6	
80–89	25.5	25.9	25.4	
90–100	2.6	2.6	2.6	
Octogenarians, %	28.1	28.4	28	0.125
Male, %	54.4	57.3	53.5	<0.001
Race, %				<0.001
White	65.3	63	66	
Black	21.4	20.9	21.5	
Hispanic	8.8	9.8	8.5	
Asian	3.6	5.3	3	
Native American	0.8	0.8	0.8	
Others/Unknown	0.1	0.2	0.1	
Body mass index, kg/m^2^	29.0 (7.8)	28.8 (7.5)	29.0 (8.0)	<0.001
Body mass index category, %				<0.001
<18.5	30.7	30.7	30.6	
18.5–24.9	28.7	30	28.3	
25–29.9	36.3	35.2	36.6	
≥30	3.6	3.4	3.6	
Missing	0.8	0.7	0.8	
Comorbidities, %				
Congestive heart failure	39	25.5	43.2	<0.001
Atherosclerotic hearth disease	41.9	33.2	44.6	<0.001
Hypertension	87.3	89.1	86.7	<0.001
Diabetes mellitus	56.8	52	58.3	<0.001
Cancer	10.2	9.8	10.3	0.005
Amputations	3.3	2.2	3.7	<0.001
Peripheral vascular disease	15.8	12.2	16.8	<0.001
CVA/TIA	11.5	9	12.3	<0.001
COPD	13.8	9	15.2	<0.001
Cause of ESRD, %				<0.001
Diabetes mellitus	44.6	41.4	45.6	
Hypertension/LVD	34.4	37.2	33.5	
Malignancy	2.6	2.7	2.6	
Cystic/Hereditary	1.3	2.1	1	
Vasculitis/secondary GN	1.2	1.4	1.2	
GN	3.7	4.8	3.4	
Interstitial nephritis/pyelonephritis	2.9	3	2.8	
Others/Unknown	9.3	7.5	9.9	
Current smoker, %	5	5.2	5	0.051
Alcohol use, %	1	0.8	1.1	<0.001
Drug use, %	0.5	0.4	0.6	<0.001
Initial modality, %				<0.001
Hemodialysis	94.3	91.2	95.3	
Peritoneal dialysis	5.7	8.8	4.7	
Primary dialysis setting, %				<0.001
Dialysis facility	93	90.5	93.7	
Home	5.9	9	5	
Skilled nursing facility	1.1	0.4	1.3	
Hemodialysis access, %				<0.001
AV access	20	28	17	
Central venous catheter	80	72	83	
Maturing AV access, among those with CVC	19	19	19	0.162
Pre-dialysis nephrology care, %				<0.001
None	24.1	21.6	24.9	
<12 months	32.2	30.9	32.6	
>12 months	30.3	36.5	28.4	
Unknown	13.4	11	14.2	
Laboratory variables from first 45-day period from date of first dialysis				
Albumin, mg/dl	3.2 (5.0)	3.4 (5.5)	3.2 (4.8)	<0.001
Albumin category, %				<0.001
<3.5	47.7	41.7	49.6	
≥ 3.5	23.8	30.5	21.7	
Missing	28.5	27.8	28.7	
Hemoglobin, g/dl	9.9 (13.0)	10.2 (17.0)	9.9 (11.5)	0.002
Hemoglobin category, %				<0.001
<11	71.4	68.7	72.2	
11–12	10.6	11.9	10.2	
>12	5.8	6.9	5.5	
Missing	12.2	12.5	12.1	
Employment Status, %				<0.001
Unemployed	12.1	11.6	12.2	
Retired due to age	63.3	64.9	62.7	
Retired due to disability	20	18.1	20.6	
Employed/others	4.7	5.4	4.5	
Access to transplant information, %	74.8	77.5	73.9	<0.001
Survival in months ^a^	23.9 (18.0)	28.0 (18.4)	22.7 (17.7)	<0.001
90-day mortality, %	11.2	7	12.5	<0.001
1-year mortality, %	30.3	20.8	33.2	<0.001
Cause of death, %				<0.001
Cardiovascular	35.6	35	35.7	
Infection	8.4	8.2	8.4	
Malignancy	3.8	4.8	3.6	
Withdrawal of dialysis	14.7	15.3	14.6	
Unknown/others	37.5	36.7	37.7	

Data are presented in mean (SD), or proportion where appropriate. CVA/TIA, cerebrovascular accident/transient ischemic attack; COPD, chronic obstructive pulmonary disease; AV, arteriovenous; CVC, central venous catheter; LVD, large vessel disease; GN, glomerulonephritis

^a^ censored on December 31, 2015

Overall, 1-year all-cause mortality was 30.3%, and 90-day mortality was 11.2%. Both 1-year mortality and 90-day mortality were higher in patients with any pre-dialysis hospitalization as compared to patients’ with no pre-dialysis hospitalization (33.2% vs. 20.8%, p<0.001 and 12.5% vs. 7.0%, p<0.001 respectively).

**Fig 2:**
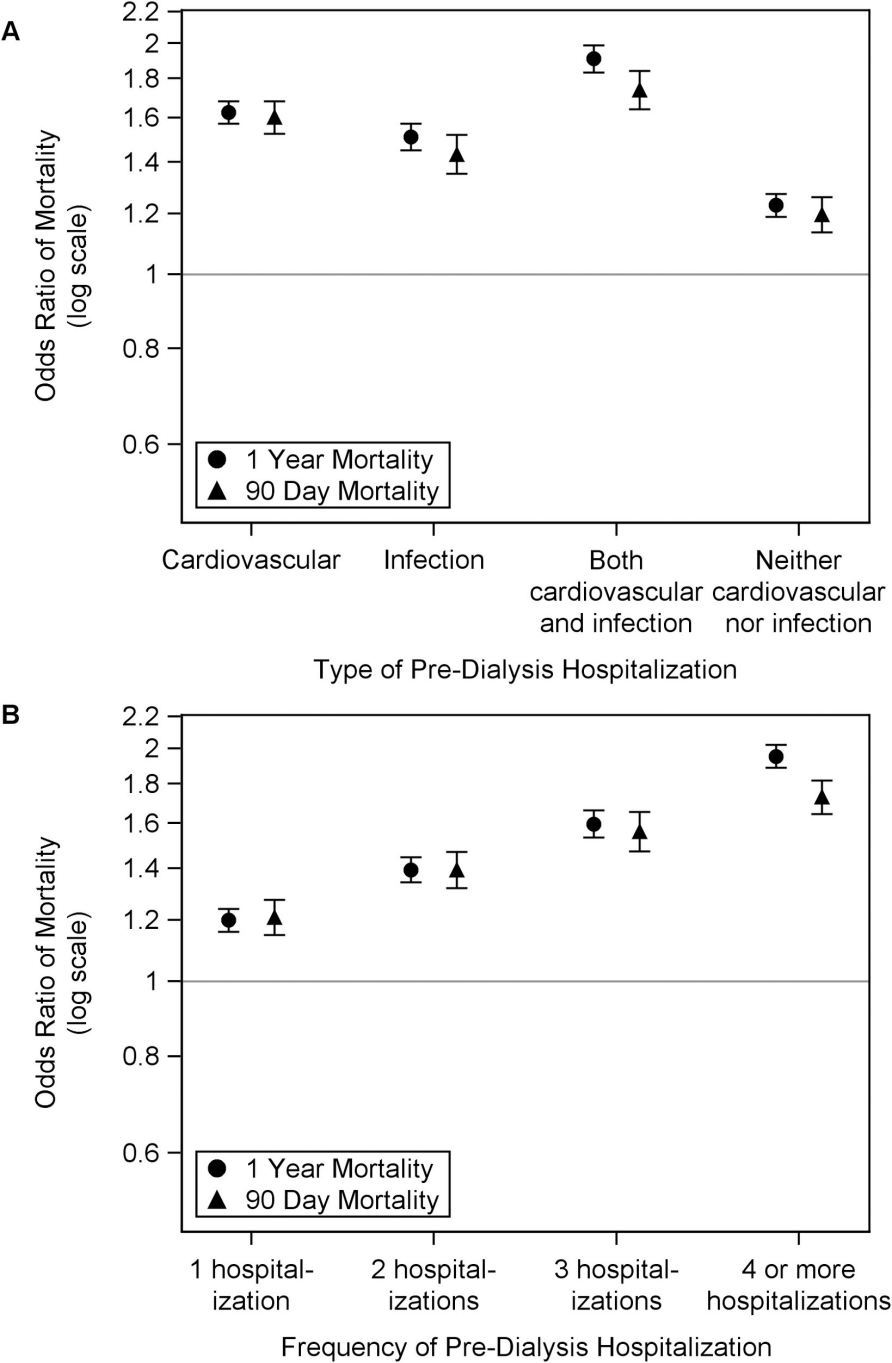
Association of pre-dialysis hospitalization with 1-year and 90-day mortality for initiation of hemodialysis among patients with ESRD. Adjusted odds ratio for 1-year mortality and 90-day mortality by (A) type of pre-dialysis hospitalization, and (B) frequency of hospitalization. (A) Compared to no pre-dialysis hospitalization, the odds ratios, fully adjusted, and 95% confidence interval for 1-year mortality in incident ESRD patients by type of hospitalization: cardiovascular, 1.63 (1.57, 1.68); infection, 1.51 (1.45, 1.57); both cardiovascular and infection, 1.91 (1.83, 1.99); and neither cardiovascular nor infection, 1.23 (1.19, 1.27); odds ratios and confidence intervals for 90-day mortality: cardiovascular, 1.60 (1.53, 1.68); infection, 1.43 (1.35, 1.52); both cardiovascular and infection, 1.74 (1.64, 1.84); and neither cardiovascular nor infection, 1.20 (1.14, 1.26). (B) Compared to no pre-dialysis hospitalization, the odds ratios, fully adjusted, and 95% confidence interval for 1-year mortality in incident ESRD patients by frequency of hospitalization: 1, 1.20 (1.16, 1.24); 2, 1.39 (1.34, 1.45); 3, 1.60 (1.53, 1.66); and 4 or more, 1.95 (1.89, 2.02); odds ratios and confidence intervals for 90-day mortality: 1, 1.21 (1.15, 1.27); 2, 1.39 (1.32, 1.47); 3, 1.56 (1.47, 1.66); and 4 or more, 1.73 (1.65, 1.82).

**Table 2** shows independent predictors of 1-year mortality in ESRD patients from a multivariable regression model. Patients with any hospitalization were at a higher risk of death within 1 year than those who did not have a pre-dialysis hospitalization. Compared with no pre-dialysis hospitalization, patients had a higher odds of dying within 1 year with cardiovascular hospitalization (OR, 1.63; 95% CI, 1.57–1.68), infection hospitalization (OR, 1.51; 95% CI, 1.45–1.57), both infection and cardiovascular hospitalization (OR, 1.91; 95% CI, 1.83–1.99) and neither-cardiovascular nor-infection hospitalization (OR, 1.23; 95% CI, 1.19–1.27) **(Fig 2A).** Additional factors associated with higher risk of 1-year mortality included presence of specific comorbidities like congestive heart failure and peripheral vascular disease, absence of pre-dialysis nephrology care, presence of nursing home history, male gender, white race, low albumin, and lack of access to transplant information. In a separate regression model, which examined the effect of multiple pre-dialysis hospitalizations, each hospitalization increased the risk of dying, independent of other covariates. Compared with no pre-dialysis hospitalization, patients had higher odds of dying within 1 year with 1 pre-dialysis hospitalization (OR, 1.20; 95% CI, 1.16–1.24); 2 pre-dialysis hospitalizations (OR, 1.39; 95% CI, 1.34–1.45); 3 pre-dialysis hospitalizations (OR, 1.60; 95% CI, 1.53–1.66) and ≥ 4 pre-dialysis hospitalizations (OR, 1.95; 95% CI, 1.89–2.02) **(Fig 2B).** The adjusted odds of 90-day mortality were also higher in patients with any type hospitalization, **(Fig 2A)** or with ≥ 1 hospitalization **(Fig 2B).**

**Table 2:** Independent variable predictors of 1-year mortality in the final regression model.

Variable^a^	Odds ratio (95% CI)	P Value
Type of hospitalization		<0.001
No hospitalization	Reference	
Cardiovascular	1.63 (1.57, 1.68)	
Infection	1.51 (1.45, 1.57)	
Both cardiovascular and infection	1.91 (1.83, 1.99)	
Neither cardiovascular nor infection	1.23 (1.19, 1.27)	
Age ≥80 years	1.64 (1.60, 1.68)	<0.001
Body mass index, kg/m^2^		<0.001
<18.5	1.84 (1.74, 1.95)	
18.5–24.9	1.35 (1.31, 1.39)	
25–29.9	1.13 (1.10, 1.17)	
>30	Reference	
Missing	1.30 (1.15, 1.47)	
Females	0.94 (0.92, 0.96)	<0.001
Race		<0.001
White	Reference	
Black	0.68 (0.66, 0.70)	
Hispanic	0.66 (0.63, 0.68)	
Asian	0.59 (0.56, 0.63)	
Native American	0.72 (0.63, 0.82)	
Unknown/Other	1.37 (1.04, 1.82)	
Poor functional status	1.43 (1.39, 1.47)	<0.001
History of nursing home	1.70 (1.64, 1.77)	<0.001
Congestive heart failure	1.32 (1.29, 1.36)	<0.001
Atherosclerotic hearth disease	1.12 (1.10, 1.15)	<0.001
Hypertension	0.75 (0.73, 0.78)	<0.001
Diabetes mellitus	0.94 (0.92, 0.97)	<0.001
Cancer	1.35 (1.30, 1.40)	<0.001
Peripheral vascular disease	1.09 (1.06, 1.12)	<0.001
COPD	1.21 (1.18, 1.25)	<0.001
Smoking history	0.91 (0.86, 0.96)	0.001
Alcohol use	1.26 (1.13, 1.40)	<0.001
Drug abuse	0.77 (0.65, 0.90)	0.002
Unemployed	0.94 (0.91, 0.98)	0.001
Albumin, mg/dl		<0.001
<3.5	1.43 (1.39, 1.48)	
≥3.5	Reference	
Missing	1.34 (1.30, 1.39)	
Hemoglobin, g/dl		<0.001
<11	0.86 (0.82, 0.90)	
11–12	0.93 (0.88, 0.99)	
>12	Reference	
Missing	0.96 (0.90, 1.01)	
Dialysis access		<0.001
Hemodialysis AV access	Reference	
Central venous catheter	1.85 (1.79, 1.91)	
Peritoneal dialysis	1.29 (1.21, 1.37)	
Cause of ESRD		<0.001
Diabetes mellitus	Reference	
Hypertension/LVD	1.08 (1.05, 1.11)	
Malignancy	2.00 (1.86, 2.14)	
Cystic/hereditary	0.72 (0.64, 0.81)	
Vasculitis/secondary GN	1.02 (0.92, 1.13)	
GN	0.83 (0.78, 0.89)	
Interstitial nephritis/pyelonephritis	0.93 (0.86, 0.99)	
Others/Unknown	1.29 (1.24, 1.35)	
Pre-dialysis nephrology care		<0.001
None	Reference	
0–12 months	0.83 (0.81, 0.85)	
>12 months	0.70 (0.68, 0.73)	
Unknown	1.11 (1.07, 1.15)	
Access to transplant information	0.76 (0.74, 0.78)	<0.001

^a^Factors included in the model but not found to be significant in incident ESRD patients included insurance coverage, amputation and CVA/TIA.

CVA/TIA, cerebrovascular accident/ transient ischemic attack; COPD, chronic obstructive pulmonary disease; AV; arteriovenous, LVD, large vessel disease; GN, glomerulonephritis

**Fig 3:**
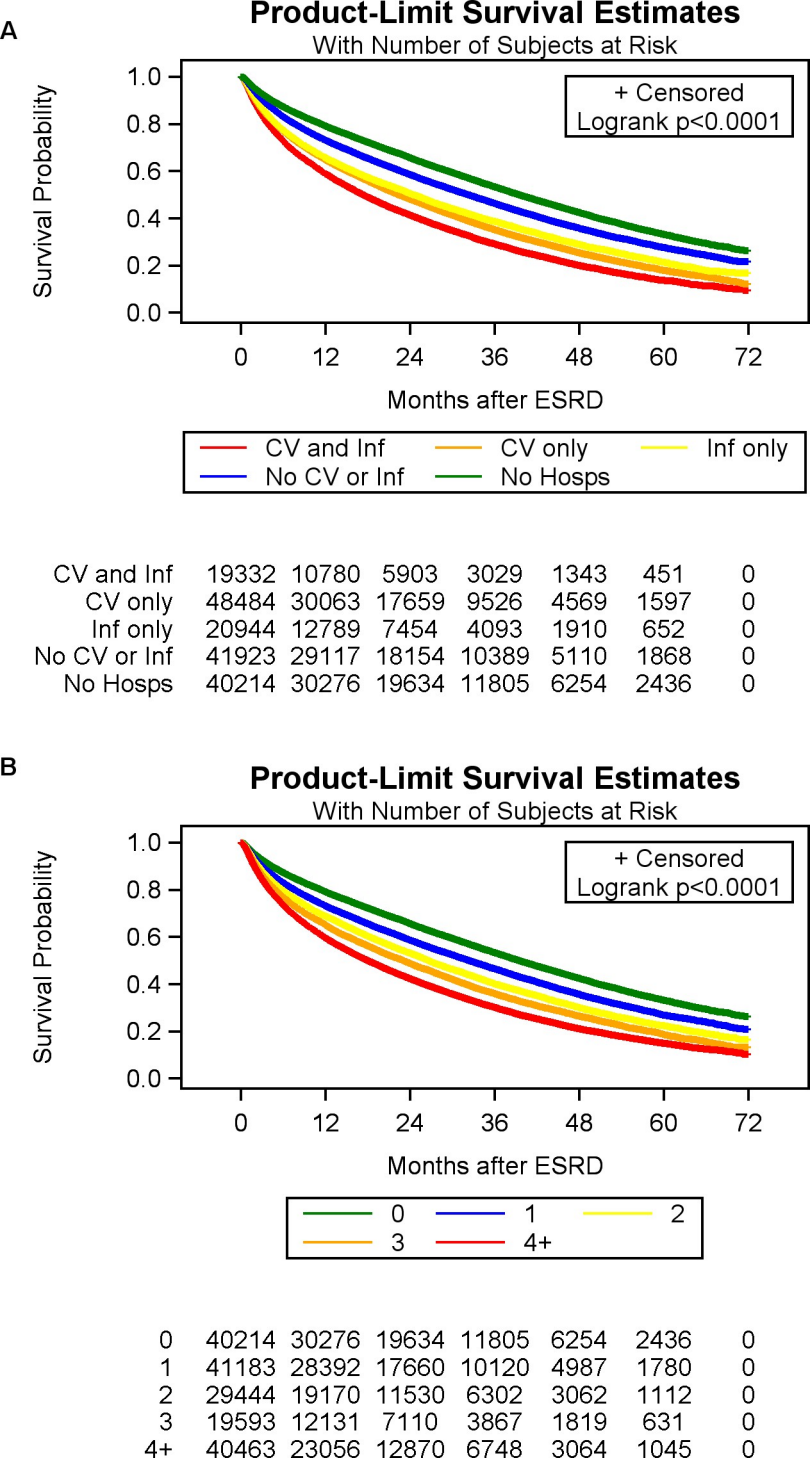
**Unadjusted Kaplan-Meier survival curves for patients with and without pre-dialysis acute hospitalization censored on 31 December 2015 by (A) type of pre-dialysis hospitalization, and (B) frequency of pre-dialysis hospitalization**.

The results of the Cox proportional hazards model for 1-year mortality were aligned with the results from the logistic regression for 1-year mortality. Compared with no pre-dialysis hospitalization, patients had a higher hazards of dying within 1 year with cardiovascular hospitalization (HR, 1.48; 95% CI, 1.44–1.52), infection hospitalization (OR, 1.41; 95% CI, 1.36–1.45), both infection and cardiovascular hospitalization (OR, 1.64; 95% CI, 1.59–1.70) and neither-cardiovascular nor-infection hospitalization (OR, 1.19; 95% CI, 1.15–1.22). In a separate Cox proportional hazards regression model, which examined the effect of multiple pre-dialysis hospitalizations, each hospitalization increased the hazard of dying, independent of other covariates. Compared with no pre-dialysis hospitalization, patients had higher odds of dying within 1 year with 1 pre-dialysis hospitalization (OR, 1.17; 95% CI, 1.14–1.21); 2 pre-dialysis hospitalizations (OR, 1.32; 95% CI, 1.28–1.36); 3 pre-dialysis hospitalizations (OR, 1.45; 95% CI, 1.41–1.50) and ≥ 4 pre-dialysis hospitalizations (OR, 1.67; 95% CI, 1.63–1.72). **Fig 3A** represents unadjusted survival curves for patients with and without pre-dialysis acute hospitalization by type, censored on 31 December 2015. **Fig 3B** represents unadjusted survival curves for patients without pre-dialysis hospitalization and those with multiple episodes of pre-dialysis hospitalization, censored on December 31, 2015.

In the study cohort, 63.9% of patients with pre-dialysis hospitalization received in-hospital dialysis initiation as compared to 56.9% with no history of pre-dialysis hospitalization. Compared with patients with no pre-dialysis hospitalization, patients had higher odds of in-hospital dialysis initiation with history of pre-dialysis cardiovascular hospitalization (OR, 1.11; 95% CI, 1.08–1.49), infection hospitalization (OR, 1.08; 95% CI, 1.04–1.12), and both cardiovascular and infectious hospitalization (OR, 1.12; 95% CI, 1.08–1.65) (Table 3).

**Table 3:** Association between inpatient dialysis initiation and pre-ESRD hospitalization.

Variable	Odds ratio
Type of hospitalization	
No hospitalization	Reference
Cardiovascular	1.11 (1.08, 1.49)
Infection	1.08 (1.04, 1.12)
Both cardiovascular and infection	1.12 (1.08, 1.65)
Neither cardiovascular nor infection	0.96 (0.93, 0.99)

Logistic model adjusted for BMI, sex, race, functional status, comorbidities, history of nursing home, employment status, history of tobacco, insurance, laboratory parameters, cause of ESRD and pre-dialysis nephrology care.

**Fig 4:**
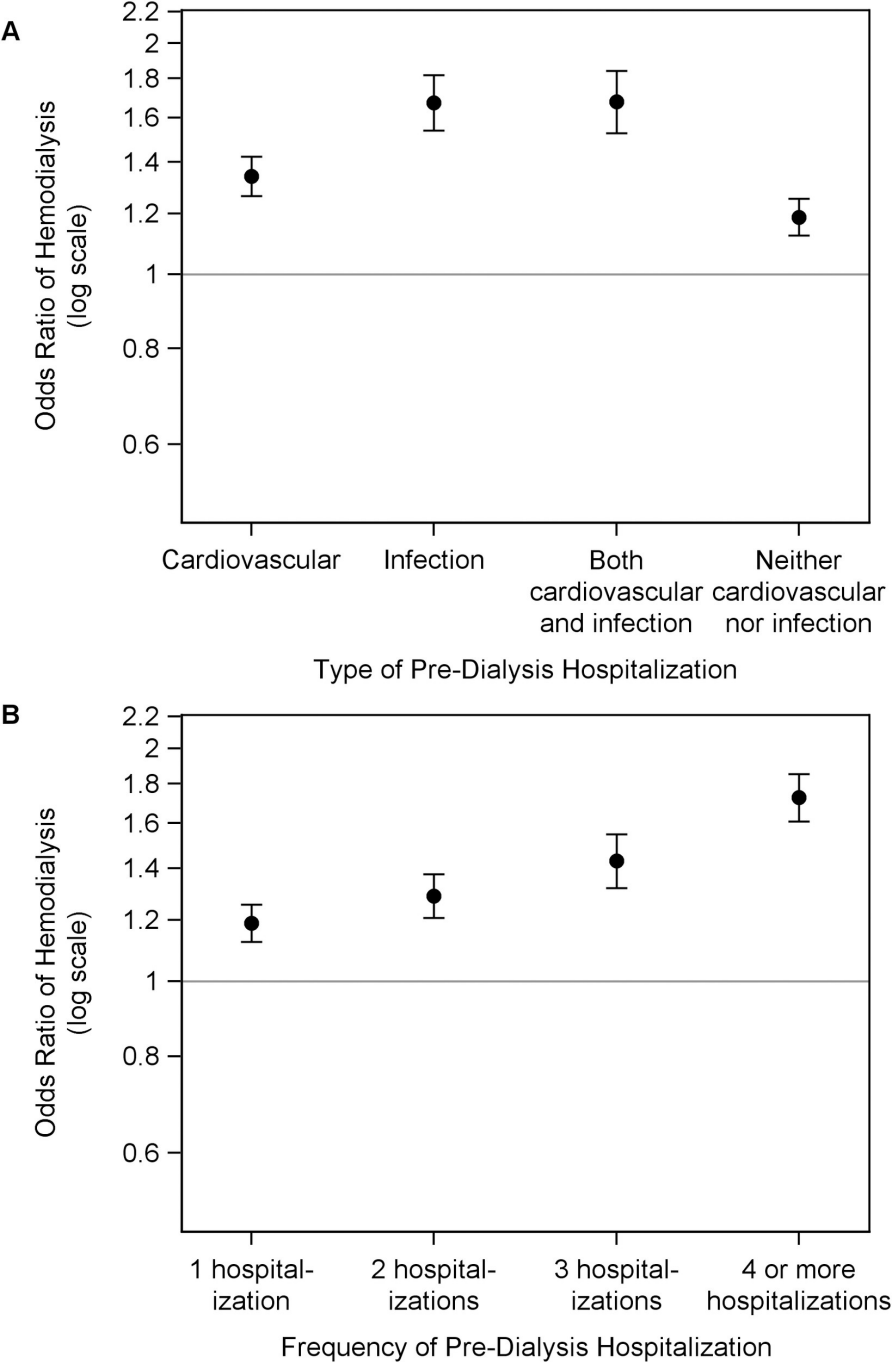
Association of pre-dialysis hospitalization with hemodialysis as the initial dialysis modality among patients with ESRD. Adjusted odds ratio for probability of hemodialysis vs. peritoneal dialysis to initiate hemodialysis by (A) type of pre-dialysis hospitalization, and (B) frequency of hospitalization. Compared to no pre-dialysis hospitalization, the odds ratios, fully adjusted, and 95% confidence interval in incident ESRD patients by (A) type of hospitalization: cardiovascular, 1.34 (1.27, 1.42); infection, 1.67 (1.54, 1.82); both cardiovascular and infection, 1.68 (1.53, 1.84); and neither cardiovascular nor infection 1.19 (1.12, 1.25); and (B) frequency of hospitalization: 1, 1.19 (1.13, 1.26); 2, 1.29 (1.21, 1.38); 3, 1.43 (1.32, 1.55); and 4 or more, 1.73 (1.61, 1.85).

Next, we looked at the secondary outcome of hemodialysis as the initial dialysis modality in patients with ESRD. Cardiovascular, infection, both cardiovascular and infection, and neither cardiovascular nor infection pre-dialysis hospitalization increased the risk of hemodialysis vs. peritoneal dialysis as the initial dialysis modality, as compared to those with no pre-dialysis hospitalization (OR, 1.34; 95% CI, 1.27–1.42; OR, 1.67; 95% CI, 1.54–1.82; OR, 1.68; 95% CI, 1.53–1.84; and OR, 1.19; 95% CI, 1.12–1.25 respectively) **(Fig 4A).** In a separate model, compared with no pre-dialysis hospitalization, patients had higher odds of using hemodialysis as the initial dialysis modality with 1 hospitalization (OR, 1.19; 95% CI, 1.13–1.26), 2 hospitalizations (OR, 1.29; 95% CI, 1.21–1.38), 3 hospitalizations (OR, 1.43; 95% CI, 1.32–1.55); and ≥ 4 hospitalizations (OR, 1.73; 95% CI, 1.61–1.85) **(Fig 4B).**

**Fig 5:**
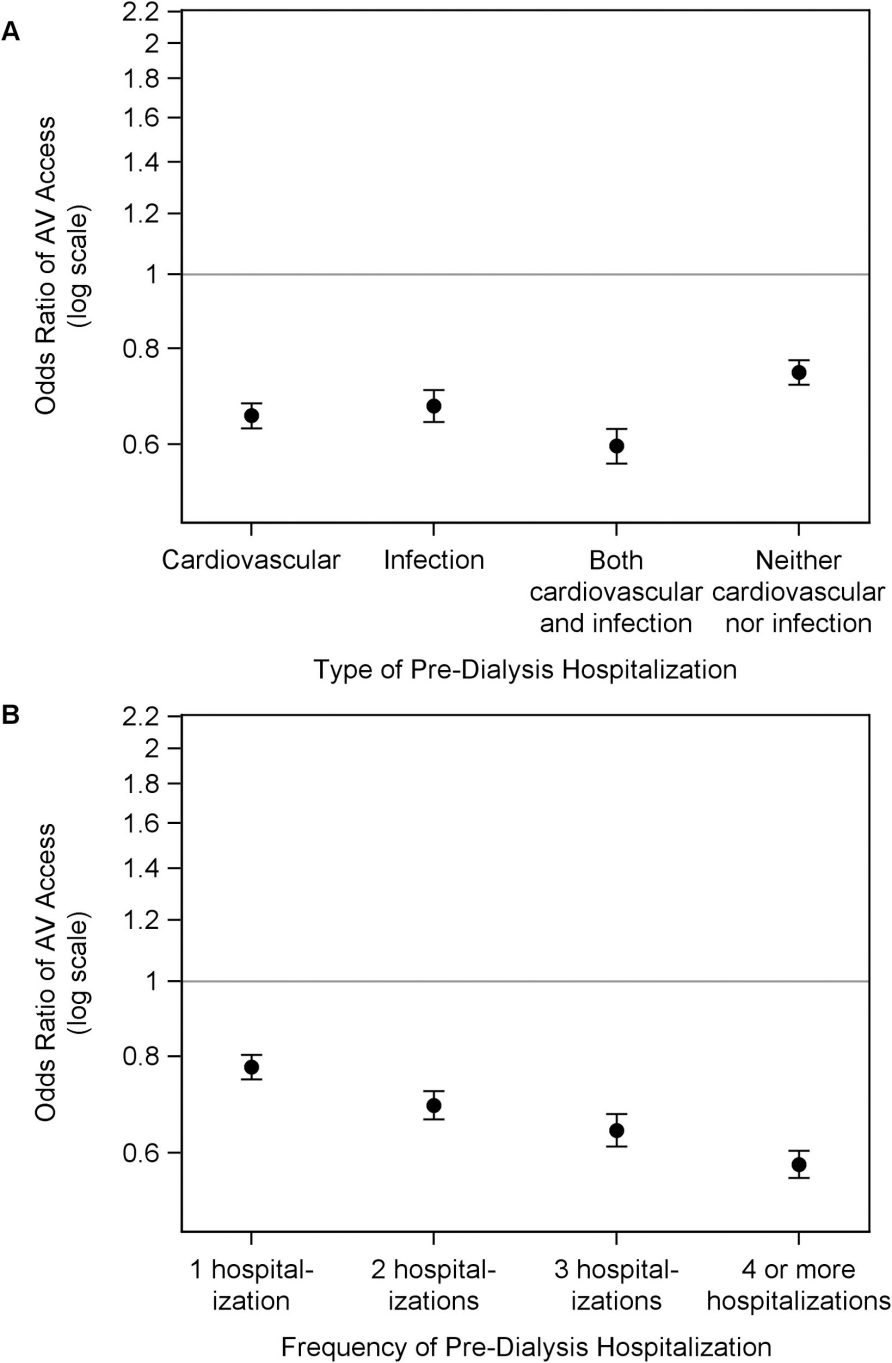
Association of pre-dialysis hospitalization with use of AV access for initiation of hemodialysis among patients with ESRD. Adjusted odds ratio for probability of AV access use vs. central venous catheter to initiate hemodialysis by (A) type of pre-dialysis hospitalization, and (B) frequency of hospitalization. Compared to no pre-dialysis hospitalization, the odds ratios, fully adjusted, and 95% confidence interval in incident ESRD patients by (A) type of hospitalization: cardiovascular, 0.65 (0.63, 0.68); infection, 0.67 (0.64, 0.71); both cardiovascular and infection, 0.60 (0.57, 0.63); and neither cardiovascular nor infection 0.75 (0.72, 0.77); and (B) frequency of hospitalization: 1, 0.78 (0.75, 0.80); 2, 0.69 (0.66, 0.72); 3, 0.64 (0.61, 0.67); and 4 or more, 0.58 (0.56, 0.60).

Finally, we examined the secondary outcome of use of AV access vs. CVC in ESRD patients with hemodialysis as the initial dialysis modality. Patients had lower adjusted odds of using AV access with pre-dialysis cardiovascular hospitalization (OR, 0.65; 95% CI, 0.63–0.68), infection hospitalization (OR, 0.67; 95% CI, 0.64–0.71), both cardiovascular and infection hospitalization (OR, 0.60; 95% CI, 0.57–0.63), and neither cardiovascular nor infection hospitalization (OR, 0.75; 95% CI, 0.72–0.77), as compared to those with no pre-dialysis hospitalization **(Fig 5A).** In a separate model, compared with no pre-dialysis hospitalization, patients had lower odds of using AV access for hemodialysis initiation with at 1 hospitalization (OR, 0.78; 95% CI, 0.75–0.80), 2 hospitalizations (OR, 0.69; 95% CI, 0.66–0.72), 3 hospitalizations (OR, 0.64; 95% CI, 0.61–0.67); and ≥ 4 hospitalizations (OR, 0.58; 95% CI, 0.56–0.60) **(Fig 5B).**

## Discussion

The present study represents a large nationwide cohort of incident ESRD patients, with linked Medicare information on each patient for two years prior to initiation of dialysis. The study finds that majority of incident dialysis patients experience frequent hospitalizations prior to ESRD initiation. More importantly, the study indicates that acute care hospitalizations prior to ESRD are not inconsequential, and that they continue to be associated with poor patient survival at one year after dialysis begins. The study also elucidates the effect of both type or frequency of hospitalization on one-year mortality, as well as on secondary outcomes of dialysis modality, and type of hemodialysis access.

Acute care hospitalizations are common among dialysis patients, and represents the single largest proportion (~ 40%) of total ESRD expenditures [17]. In an effort to improve quality of care and resource utilization in ESRD patients, studying the causes or outcomes of hospitalizations among these patients is an area of national priority. Typically, prevalent ESRD patients experience an average of two hospital stays per year; and the top two causes of hospitalization include cardiovascular events or infection related illnesses [18–20]. There are relatively fewer studies examining hospitalizations in patients with advanced CKD, prior to initiating dialysis. Holland et al looked at the acute hospitalizations in the pre-ESRD period in a small cohort (n = 362) of CKD patients; 57.4% required hospitalization prior to dialysis initiation, and one-third of these pre-dialysis admissions were due to cardiovascular causes [21]. Mix et al studied in a retrospective cohort, similar to our study design, the frequency of hospitalizations among 109, 321 patients with CKD who reached ESRD. The mean hospitalization rate was 134 hospitalizations/1,000 patient-months at risk, and rates of hospitalization increased with progression of kidney disease [10]. However, despite the frequent need for hospitalizations across the continuum of CKD progression, there are no studies that have examined the impact of pre-dialysis hospitalization on outcomes after initiation of dialysis.

In the present study, we found that about three fourths of patients with incident ESRD experienced at least one hospitalization during the 2-year pre-ESRD period, with majority of them involving cardiovascular causes. The data also indicates that more than half of the patients were hospitalized two or more times prior to incident dialysis. This high burden of hospitalizations demonstrates the high resource utilization required in patients with advanced kidney disease before they get initiated on dialysis. Patients experiencing pre-ESRD hospitalizations had poorer indicators of health status at the time of dialysis initiation; for instance, compared to those without hospitalizations, patients with pre-dialysis hospitalization had lower serum albumin and hemoglobin levels at the time of incident dialysis. Surprisingly, hospitalized patients were also less likely to have received pre-ESRD nephrology care prior to incident dialysis. It can be speculated that perhaps hospitalizations prior to ESRD could have higher probability of receiving nephrology care if these patients had either advanced CKD or suffered acute worsening of their renal function. However, studies have shown that trajectories of renal function loss are very variable in the two-year period prior to ESRD [22]. It is also plausible that acute hospitalization events undermine the need for nephrology consults, and these patients are more likely to see a primary care physician that a nephrologist after their discharge who have further shown to less recognize and recommend specialist care for progressive CKD [23, 24]. These observations raises the question whether CKD patients experiencing hospitalization in the pre-ESRD period will benefit from closer monitoring by a health care provider during convalescence, transitional model of care units, or other systems-based opportunities to improve dedicated pre-ESRD care [25].

Mortality after initiation of ESRD is considered an important metric for comparing quality of care across the dialysis industry and is linked to reimbursements. Typically, the standardized mortality rates for dialysis patients are estimated based on characteristics at the time of dialysis initiation [5, 26]. In the present study, as compared to no pre-dialysis hospitalization, a history of cardiovascular and infection related pre-dialysis hospitalization increased the risk of death by 74% at 90 days and by 91% at 1-year. Additionally, there was a graded increase in mortality with increasing frequency of pre-dialysis hospitalizations. The frequency of pre-dialysis hospitalizations might be a useful measure for risk stratifying patients initiating dialysis or considering dialysis initiation. For example, if a patient knew that if they experienced 4 or more hospitalizations in the previous 2 years, their odds of dying within the first 90 days of dialysis initiation was 73% higher, it might lead them to consider conservative management instead. In addition, CMS might find this data useful for case mix adjustment in the Prospective Payment System (PPS). Since about two-third of incident ESRD patients experienced a pre-dialysis hospitalization, reducing these hospitalizations will require multi-level changes to CKD care, as well as care of other conditions like pre-dialysis vascular access planning. We also noted significant racial differences with the risk of dying. In minorities, the risk of death was 60–70% lower as compared to white patients, similar to findings reported in prior literature [27, 28]. Since our study cohort only consisted of patients who survived following initiation of dialysis, it is possible that sicker white patients made it to dialysis more frequently compared to minorities due to better access of care and more aggressive interventions.

This study has several limitations. First, the data were derived from an administrative dialysis dataset, and the pre-dialysis information was made available from Medicare claims files. However, such limitations are inherent in de-identified national cohorts, in the absence of longitudinal datasets with reliable patient level information. Second, the hospitalization information was only analyzed for the period of two calendar years prior to incident ESRD, and for only those patients who were primarily Medicare beneficiaries during that entire time period. This may lead to over representation of older patients in our cohort and may not remain generalizable to broader ESRD population. Medicare claims data, as has been reported in other similar studies, can have low sensitivity, and potentially can lead to misclassification of the types of hospitalization. To mitigate this weakness, we used validated codes for primary admission diagnoses, and we analyzed our data both by cause as well as frequency of hospitalizations. We ascertained the comorbidities from the CMS-2728 form instead of Medicare claims, which remains another limitation of the study. Finally, in the present design, our study could not examine the association of hospitalization with outcomes in advanced CKD, but was able to determine the association of hospitalization with future outcomes among those CKD patients only who survived to initiate dialysis.

Despite these limitations, these findings have several important implications for ESRD care. ESRD initiation is a life-changing event for a patient, which is also associated with changes in providers, healthcare systems, and health insurance status. During this transition of care period, it is common to either not have access to or under-recognize important factors during the pre-dialysis period that may impact post-dialysis care. Our observations from a national cohort point out that both the type and frequency of acute care hospitalizations prior to dialysis initiation are of prognostic significance. We found that about 75% of patients had occurrence of pre-dialysis hospitalization. Although these hospitalizations could not necessarily be avoided, as they likely occur for some acute illness, the observation still allows us to speculate about how this information may lead to future improvements. One possibility is that better pre-ESRD care can potentially reduce the risk of pre-ESRD hospitalizations, and this can be associated with improved outcomes. Additionally, the study also shows that pre-ESRD hospitalization may be a lost opportunity to involve nephrology care after discharge, and efforts to enhance longitudinal follow up to nephrology specialty could potentially impact ESRD outcomes. From the standpoint of comparing the quality of dialysis care; efforts should be made to incorporate type or frequency of pre-dialysis hospitalization as an important risk factor of mortality.

## Conclusion

In conclusion, the majority of the incident ESRD patients experience at least one hospitalization prior to initiating dialysis. Both the cause and frequency of hospitalization during the pre-ESRD period significantly increases one-year dialysis mortality. Additionally, pre-ESRD hospitalization is also associated with greater risk of initiating hemodialysis versus peritoneal dialysis; and among hemodialysis patients, it lowers the odds of presence of AV access at dialysis initiation. The study emphasizes that both healthcare providers and dialysis providers need to consider that pre-dialysis acute care utilization can continue to impact outcomes after initiation of dialysis. Whether this presents an opportunity to either intervene prior to dialysis, or to customize care after dialysis begins requires further investigation.

## Supporting information

S1 Table ICD9 codes for cardiovascular causes and infectious causes of acute hospitalizations. (DOCX)
