# Supplementary material for: Pre-dialysis acute care hospitalizations and clinical outcomes in dialysis patients
Source: PLoS One. 2019 Jan 16;14(1):e0209578. doi: 10.1371/journal.pone.0209578 (PMC6334901; doi:10.1371/journal.pone.0209578)
Supplement: S1 Table — (DOCX) [file pone.0209578.s001.docx]

Supplement 1

| **Cardiovascular causes** | **ICD 9 Code** |
| --- | --- |
| Congestive heart failure, Cardiomyopathy | 276.6, 398.91, 402.01, 402.11, 402.91, 404.01, 404.03, 404.11, 404.13, 404.91, 404.93, 425, 428, 518.4 |
| Ischemic heart disease | 410-414 |
| Conduction disorders and dysrhythmias | 426, 427, 798 |
| Cerebrovascular disease | 430-438 |
| Circulatory disease | 250.7, 440-456, 458, 459 |
| Other cardiac disease | 394-398, 415-420, 422-424, 429 |
| **Infectious Causes** | **ICD 9 Codes** |
| Sepsis | 038, 790.7 |
| Access related infection | 567, 996.62, 996.68, 999.31 |
| Endocarditis | 036.42, 421 |
| Respiratory tract infection | 011, 012, 033, 034, 460-466, 473, 480-488, 490, 491.1, 494, 510, 511, 513, 518.6, 519.01 |
| Skin/bone/joint infection | 015, 035, 036.82, 680-686, 706.0, 711, 730, 996.66, 996.67 |
| Gastrointestinal infection | 001-009, 014, 567, 569.5 |
| Central nervous system infection | 013, 036.0, 036.1, 036.2, 036.81, 045-049, 062-064, 320-326 |
| Fungal infection | 112, 117 |
| Genitourinary infection | 016, 590, 595-595.4, 597, 598.0, 599.0, 601-601.9, 604, 607.1, 607.2, 608.0, 608.4, 614, 616.1, 616.3, 616.4, 616.8, 670, 996.64, 996.65 |
| Other infection | 010, 017, 018, 020-027, 030-032, 036.3, 036.4, 036.89, 036.9, 037, 039-042, 050-059, 060, 061, 065, 066, 070-079, 080-088, 090-099, 100-139, 254.1,331.81, 373.0, 373.1, 373.2, 382.0, 382.1, 382.2, 382.3, 382.4, 383.0, 386.33, 386.35, 388.60, 390-393, 422.0, 422.91, 422.92, 422.93, 472-475, 478.21, 478.22, 478.23, 478.24, 478.29, 522.5, 522.7, 527.3, 528.3, 540-542, 566, 572.0, 572.1, 573.1, 573.2, 573.3, 575.0, 575.1, 611.0, 790.8, 998.5, 996.60, 996.61, 996.63, 996.69, 997.62, 999.3 |
